# Physicochemical Characterization of a Thermostable Alcohol Dehydrogenase from *Pyrobaculum aerophilum*

**DOI:** 10.1371/journal.pone.0063828

**Published:** 2013-06-05

**Authors:** Annalisa Vitale, Natasha Thorne, Scott Lovell, Kevin P. Battaile, Xin Hu, Min Shen, Sabato D'Auria, Douglas S. Auld

**Affiliations:** 1 National Center for Advancing Translational Sciences, Bethesda, Maryland, United States of America; 2 Institute of Protein Biochemistry, NRC, Via Pietro Castellino, Naples, Italy; 3 Del Shankel Structural Biology Center, Protein Structure Laboratory, University of Kansas, Lawrence, Kansas, United States of America; 4 Industrial Macromolecular Crystallography Association Collaborative Access Team, Hauptman-Woodward Medical Research Institute, Argonne, Illinois, United States of America; Griffith University, Australia

## Abstract

In this work we characterize an alcohol dehydrogenase (ADH) from the hyperthermophilic archaeon *Pyrobaculum aerophilum* (PyAeADHII). We have previously found that PyAeADHII has no activity when standard ADH substrates are used but is active when α-tetralone is used as substrate. Here, to gain insights into enzyme function, we screened several chemical libraries for enzymatic modulators using an assay employing α-tetralone. The results indicate that PyAeADHII activity in the presence of α-tetralone was inhibited by compounds such as flunarizine. We also examined metal coordination of the enzyme in solution by performing metal substitution of the enzyme-bound zinc (Zn^2+^) with cobalt. The solution-based absorption spectra for cobalt substituted PyAeADHII supports substitution at the structural Zn^2+^ site. To gain structural insight, we obtained the crystal structure of both wild-type and cobalt-substituted PyAeADHII at 1.75 Å and 2.20 Å resolution, respectively. The X-ray data confirmed one metal ion per monomer present only at the structural site with otherwise close conservation to other ADH enzymes. We next determined the co-crystal structure of the NADPH-bound form of the enzyme at 2.35 Å resolution to help define the active site region of the enzyme and this data shows close structural conservation with horse ADH, despite the lack of a catalytic Zn^2+^ ion in PyAeADHII. Modeling of α-tetralone into the NADPH bound structure suggests an arginine as a possible catalytic residue. The data presented here can yield a better understanding of alcohol dehydrogenases lacking the catalytic zinc as well as the structural features inherent to thermostable enzymes.

## Introduction

Alcohol dehydrogenases (ADHs; EC 1.1.1.1) are enzymes widely distributed in all living organisms (archaea, bacteria, fungi, plants and animals) [1] and play an important role in a broad range of physiological processes (e.g. alcohol and alkane metabolism, cell defense towards exogenous alcohols and aldehydes) [2]. There are on-going efforts to structurally and functionally characterize ADHs from hyperthermophilic bacteria (optimal growth above 100°C). These ADHs are found to display extreme stability at high temperature, high pressure, and high concentrations of chemical denaturants, while also demonstrating broad substrate specificity [3]. The ADH enzyme family can catalyze the inter-conversion of a large number of compounds including branched and cyclic alcohols, aliphatic and aryl aldehydes, linear, branched and cyclic ketones and aliphatic and aryl-keto esters. Many of the functional groups within these structures are also found in chemical libraries employed in high-throughput screening (HTS) – large chemical libraries used to identify leads for drug discovery and for studying chemical biology. This suggests that screening ADH enzymes against HTS chemical libraries could yield both inhibitors and substrates of the enzyme.

Enzymes such as ADH have the capability of catalyzing chemo-, stereo- and regio-selective reactions to produce enantiomerically pure products [4]. The thermostable feature of some of these enzymes makes them commercially more attractive than their mesophilic counterparts because the improved enzyme stability offers considerably more potential for a range of biotechnological applications in food, pharmaceutical and fine chemical industries [5], [6]. Thermostable structures can also make enzymes more amenable to specific mutations aimed at designing enzymes that catalyze unique chemical reactions.

In the present work, our attention was focused on a thermostable ADH from the hyperthermophilic archaeon *Pyrobaculum aerophilum* (PyAeADHII) [7]. This ADH is characterized as belonging to the medium-chain dehydrogenase/reductase (MDR) superfamily, with a size of 330 residues and a structural Zn^2+^ binding site made up of four closely spaced cysteine residues localized in a lobe at the periphery of the catalytic domain [8]. However, previous studies have revealed that PyAeADHII has peculiar characteristics because the enzyme lacked activity on most standard compounds used to test the activity of ADHs, and was active only when α-tetralone was used as a substrate [9]. Moreover, sequence alignment of PyAeADHII with sequences of well characterized ADHs, such as horse liver ADH (HLADH 6ADH_B) [10], *Saccharomyces cerevisiae* ADH (YADH CAA91579) [11], [12] and *Sulfolobus solfataricus* ADH (SsADH CAA87591) [13] showed that the PyAeADHII lacks key residues involved in the catalytic Zn^2+^binding (e.g. Cys-46, His-67 and Cys-174 in HLADH; these are found as Asn-39, Ser-61, and Ile-147 in PyAeADHII) and the key residue involved in the catalytic event, corresponding to Ser-48 in YADH, Ser-40 in SsADH and Thr-45 in HLADH.

To identify potential probes acting as either substrates, inhibitors, or activators of PyAeADHII, we screened libraries of annotated low-molecular weight compounds (drugs or drug-like compounds) using quantitative HTS (qHTS) – a paradigm in which compounds in large chemical libraries are rapidly tested for activity in an assay at multiple concentrations, thereby yielding concentration-response curves (CRCs) for every compound, and greatly reducing the frequency of false positives and false negatives [14]. While we were unable to confirm substrates or activators of the enzyme, the screen did identify a series of chemically-related inhibitors with weak potency against the enzyme. Inhibition of the enzyme by the compounds identified in the HTS was confirmed using spectrophotometric assays, validating that these inhibitory compounds are the first compounds known to inhibit PyAeADHII. Additionally, we decided to undertake biophysical studies to further characterize the thermostablity, metal coordination, and NADPH binding of this MDR ADH. For these studies, we substituted the PyAeADHII enzyme-bound Zn^2+^ with cobalt and measured the spectral properties of the enzyme in solution, we obtained the crystal structure of both wild-type (PyAeADHII-WT) and cobalt-bound (PyAeADHII-Co) at 1.75 Å and 2.2 Å resolution, respectively, and determined the co-crystal structure of the NADPH-bound form (PyAeADHII-NADPH) of the enzyme at 2.35Å resolution. The structural studies show strict homology to other mesophilic ADHs despite the high thermostability and the lack of a catalytic Zn^2+^ ion. Examination of the NADPH-bound structures points to the catalytic region in PyAeADHII and an arginine as a possible residue involved with catalysis.

## Materials and Methods

### Spectrophotometric assay in standard conditions

The wild-type PyAeADHII was cloned and purified as described elsewhere [9]. PyAeADHII activity was assayed at 37°C by measuring the change in absorbance of NADPH at 340 nm, using a Cary 1E spectrophotometer equipped with a Peltier effect-controlled temperature cuvette holder. The standard assay for the reduction reaction of α-tetralone was performed by adding 2.6 μM final concentration of the enzyme to a preheated assay mixture containing 3 mM substrate (α-tetralone; Sigma-Aldrich #T19003, Lot S28914 or from Tokyo Chemical Industry #T0134), 0.3 mM NADPH (Sigma-Aldrich #N5130) in 50 mM sodium phosphate (pH 7.5) [15]. The protein concentration was determined by spectrophotometry (Cary 1E, Varian). The molar extinction constant for PyAeADHII was taken as E_280_  = 34,630 M^−1^ cm^−1^ and molecular weight of PyAeADHII is approximately 38.5 kDa. This value is calculated based on fact that the recombinant PyAeADHII contains an extraneous N-terminus that is approximately 30 residues in length (containing a His-tag) and the full-length PyAeADHII contains 360 residues, 12 of which are tyrosines and three are tryptophans. Under the conditions described above the enzyme showed a Specific Activity value of 0.08 U/mg. One unit of PyAeADHII is defined as the enzyme amount required to oxidize 1 μmole of NADPH per min at 37°C, on the basis of an absorption coefficient at 340 nm for NADH of 6.22 mM^−1^cm^−1^.

Interestingly, we found that some samples of α-tetralone purchased from Sigma-Aldrich and Tokyo Chemical Industry appeared to contain small amounts of contaminating compounds (as determined by mass spectrometry) that effectively inhibited PyAeADHII activity. These contaminants needed to be removed by HPLC in order to isolate pure α-tetralone.

### Development of a PyAeADHII assay for HTS

An HTS assay was developed that utilized the fluorescence of NADPH (λ_Ex_ 340 nm/λ_Em_ 450 nm), a cofactor consumed in the reduction of α-tetralone by *PyAeADHII* [9], as a read-out of enzyme activity. Compounds that inhibit PyAeADHII would be expected to decrease NADPH consumption by the enzyme, and thus the fluorescence at 450 nm would be greater than uninhibited enzyme. Conversely, activators of PyAeADHII would increase consumption of NADPH, leading to decreased fluorescence at 450 nm relative to the control reaction (enzyme, NADPH, α-tetralone, and DMSO).

Previous experiments identified α-tetralone as a substrate of PyAeADHII [9]. These experiments were conducted at 70°C using a spectrophotometer. Since the detector used to measure NADPH fluorescence in high-throughput format (ViewLux plate reader, PerkinElmer, Waltham, MA) did not have the capacity to maintain this temperature, and because the detector did not have the capacity to add reagent (thus requiring time for manual loading of the assay plate after reagent addition and reaction initiation), it was necessary to optimize the temperature that could be used to initiate the reaction in parallel to optimizing the amount of enzyme that would give sufficient activity at this temperature. In addition, as the temperature of the assay plate would decrease to room temperature over time in the detector, it was not possible to perform a kinetic read for the HTS assay, and thus it was necessary to determine a single time point at which to immediately read the plate – a time point in which there was <50% NADPH consumption. Thus, prior to the HTS, optimization assays were performed in which multiple concentrations of PyAeADHII were tested, at different incubation temperatures, for different incubation times, with 3 mM α-tetralone and 0.3 mM NADPH, to determine assay conditions amendable to a HTS.

Initially, different concentrations of PyAeADHII were tested for activity at 25°C, 37°C, and 45°C using a spectrophotometer to measure NADPH depletion (A_340_) over time, and then confirmed using the fluorescence-based assay (all assays: 3 mM α-tetralone and 0.3 mM NADPH). The temperature chosen to run the fluorescence-based assay was 37°C, as the reaction rate proceeded slow enough to allow time for the assay plate to be manually loaded into the detector, but there was still enough activity to reliably measure NADPH consumption after a 20 minute incubation with substrate. Although the specific activity of the enzyme is very low at this temperature (0.08 U/mg; [9], relatively low concentrations (µM) of enzyme (e.g. low compared to the mM substrate concentrations used) could be used to further optimize the assay in 1536-well plates. Based on the results from these assays (data not shown), a final concentration of enzyme at 2.6 µM was chosen for subsequent assays at 37°C.

### The PyAeADHII assay for qHTS

Kalypsys (San Diego, Ca) black polystyrene solid bottom 1,536- well plates were used as assay plates. Polypropylene plates were used as 1,536- well compound plates. Compounds from two different chemical libraries were screened (compound number in each collection is indicated in parentheses): the Sigma- Aldrich LOPAC collection (1,280) and Tocris/TimTec (1,395; Bristol, UK). These libraries were prepared as 7-point titrations that were serially diluted 1:5 in dimethyl sulfoxide (DMSO) to establish a concentration-response series starting from 10 mM stock concentration to 0.64 µM. Each compound plate could contain up to 1,408 compound samples located in columns 5–48 with the first four columns of each 1,536- well plate reserved for control compounds. The wild-type PyAeADHII was cloned and purified as described elsewhere [9]. In the assay, the control in the first column was 6 µL of 0.3 mM NADPH (final concentration in the assay; no enzyme control), in the second column, 6 µL of the reaction mix with substrate (0.3 mM NADPH and 3 mM α-tetralone, to test for fluorescent interference of α-tetralone, and serving as a control for the maximal fluorescent signal possible from experimental wells), in the third column 23 nL of DMSO, 0.3 mM NADPH, 3 mM α-tetralone, and 2.6 µM ADH was used to determine basal activity of the enzyme in the presence of the DMSO vehicle. For columns 5–48, which would be treated with experimental compounds, three microliters/well of 2× PyAeADHII- buffer solution (5.2 µM ADH, 50 mM Sodium phosphate pH 7.5) was dispensed into 1,536-well assay plates with an Aurora discovery BioRAPTR Flying Reagent Dispenser (FRD; Beckton- Dickenson, Franklin Lakes, NJ). Using a Kalypsys pin tool equipped with 1,536-pins, 23 nL of compound solution was transferred to the assay plate, resulting in a final DMSO concentration <0.5%. The final compound concentrations in the final assay volume thus ranged from 2.4 nM to 38 µM. After adding compounds with the pin tool, the plates were initially read with the ViewLux plate reader (Perkin Elmer, Waltham, MA), using excitation wavelength of 340 nm and emission of 450 nm (15,000 excitation energy, 5 s exposure time, 1x binning) to identify any compounds that were fluorescent at these wavelengths. Following the pre-read of the plates, the reaction was initiated upon addition of three microliters/well of substrate buffer-solution (0.3 mM NADPH, 3 mM α-tetralone, 50 mM Sodium phosphate pH 7.5) which was added using the FRD, to yield a final reaction volume of 6 µL/well. After 20 mins of 37°C incubation, fluorescence was detected with the ViewLux plate reader (Perkin Elmer, Waltham, MA), using an excitation wavelength of 340 nm and emission of 450 nm, 15,000 excitation energy, 5 s exposure time, 1× binning.

Concentration-response curves (CRCs) were fitted to the data allowing assignment of CRC classes that correspond to the efficacy and potency of the compounds, the quality of the curve fits (*r* ^2^) derived from the Hill equation, and the number of asymptotes to the calculated curve [16], [17]. Based on this analysis, CRCs were organized into four categories, defined as follows: Class 1a curves were well fit (*r* ^2^ ≥0.9), showed a full response (efficacy >80%), and exhibited upper and lower asymptotes. Class 1b curves were the same as Class 1a except the efficacy value (30–80%) indicated a full but shallow curve. Class 2 curves were incomplete; they contained only one asymptote and were divided into two subclasses. Class 2a had a good fit (*r* ^2^ ≥0.9) and a sufficient response (efficacy >80%) to calculate an inflection point, whereas Class 2b characterized a weaker response (efficacy <80% and *r* ^2^ <0.9). Class 3 curves displayed activity only at the highest tested concentration with efficacy >30%. Class 4 assignments were titrations with insufficient (efficacy <30%) or no response and are hereafter referred to as inactive. Hence, the library in its entirety was defined as either active (Class 1–3) or inactive (Class 4) [16].

### Spectrophotometric assays to confirm enzyme inhibition

Spectrophotometric assays were used as orthogonal assays to confirm inhibition of PyAeADHII by flunarizine dihydrochloride and other inhibitory compounds identified in the HTS assay. These assays were performed as described above in the presence of 80 µM of inhibitor. The majority of compounds were assayed at 37°C with 2.6 µM PyAeADHII, however flunarizine dihydrochloride inhibition was also determined at 70°C (specific activity 4.75U/mg) with 260 nM PyAeADHII.

### Bacterial growth in the presence of cobalt to produce cobalt-bound PyAeADHII

To substitute the structural Zn^2+^ site with cobalt, the enzyme was expressed in the presence of cobalt. Bacterial growth was performed in minimal media M63 (100 mM potassium phosphate pH 7.0, ammonium sulfate 15 mM, magnesium sulfate 1 mM, 0.4% glycerol and 1.8 nM iron sulfate heptahydrate). The starting condition of bacterial growth to obtain the cobalt complex of PyAeADHII was the same as that used to obtain the wild-type PyAeADHII. However, at the time of induction, the bacterial growth was centrifuged to eliminate the LB media and twice the cellular pellet that was retained was re-suspended in 200 mL M63 media. After all components were mixed, approximately 50 g of Chelex resin (BioRad #142–1253) was added to 1L of media to remove Zn^2+^from the media. After one hour, the media was filtered and sterilized using a Stericap Millipore 0.22 µm filter (UFC5011008). The cells were then re-suspended in the Zn^2+^-free M63 media and allowed to grow for about 20 minutes in the shaker at 37°C. PyAeADHII expression was then induced with the addition of IPTG (isopropylβ-D-thio-galactopyranoside; Sigma-Aldrich #I5502) and CoSO_4_ to a final concentration of 0.5 mM and 10 µM, respectively. The growth of the bacterial culture continued for 18 hours at 37°C to allow over-expression of the putative Co-complex of PyAeADHII. The purification protocol used was identical to that of wild-type PyAeADHII.

### Crystallization, Data Collection and Structure Solution

Crystallization screening for all protein samples was conducted using Compact Jr. (Emerald biosystems) sitting drop vapor diffusion plates at 20°C. All X-ray diffraction data were at the Advanced Photon Source IMCA-CAT beamline 17ID using a Dectris Pilatus 6 M pixel array detector. Intensities were integrated using XDS [18] and the Laue group determination and data scaling were conducted with Aimless [19] which indicated that *P* 2/m was the correct Laue class and the likely space group was *P*2_1_. All structure refinement and manual model building with Phenix [20] and Coot [21] respectively. Structure validation was carried out using Molprobity [22] and figures were prepared using the CCP4 mg package [23].

### Wild-type PyAeADHII (ADH-WT)

Wild–type recombinant PyAeADHII (described above and in Vitale et al., [9]) was concentrated to 15.0 mg/mL in 250 mM NaCl, 50 mM Na Phosphate pH 7.5, and 125 mM imidazole was screened for crystallization. Plate shaped crystals were obtained in 1–2 days from the Wizard 2 screen condition #32 (Emerald biosystems, 20% (w/v) PEG-1000, 100 mM Tris pH 8.5) using 0.5 µL of protein and 0.5 µL of crystallization solution equilibrated against 100 µL of the latter at 20^o^ C. Single crystals were transferred to a drop containing 80% crystallization solution and 20% PEG-400 before freezing in liquid nitrogen for data collection. The Matthew's coefficient (Vm = 2.43, 49.4% solvent) suggested that there were 4 molecules in the asymmetric unit. An X-ray fluorescence scan indicated that zinc was indeed present in the crystals of the wild type PyAeADHII. Structure solution was carried out by the SAD phasing method with Shelx C/D/E [24] using zinc anomalous data to a resolution of 2.3 Å collected at a wavelength of 1.28190 Å. Four zinc sites were located with ShelxD and phasing with ShelxE yielded the following results: pseudo-free CC  = 50.79%/estimated mean FOM  = 0.482 for the original and pseudo-free CC  = 72.80%/estimated mean FOM  = 0.700 for the inverted substructures respectively. Therefore, the inverted phases were used from this point forward. A protein backbone model was obtained using the auto-tracing routine in ShelxE which produced a Cα model that was approximately 82% complete. The model was improved further with ARP/wARP [25] followed by structure refinement and manual model building. To confirm that the metal sites were indeed Zn ions, a low energy remote data set was collected using the same crystal at λ = 1.2480 Å. No peaks were observed in the anomalous difference map when using data collected at this wavelength indicating that the metal assignment was correct. A third data set was collected at λ = 1.0000 Å which was used for refinement of the final structure (PDB: 4JBG).

Large difference density peaks (Fo-Fc) greater than 5σ was observed near Arg-323 of chains B, C and D which was originally modeled as water molecules. However, following refinement this site was covered with positive electron density (Fo-Fc) at a 5σ contour level indicating an underestimation of electrons. Therefore, Cl^-^ ions were modeled at these sites and no residual difference density was observed following refinement. In addition, electron density was observed at sites near Arg-197 on chains B and D that was consistent with phosphate ions. Disordered side chains were truncated to the point where difference electron density could be observed.

### Crystallization and Data Collection of Cobalt bound PyAeADHII (ADH-Co)

A putative cobalt complex of PyAeADHII concentrated to 10.0 mg/mL in 500 mM NaCl, 50 mM NaPhosphate pH 7.5, 250 mM Imidazole was screened for crystallization. Plate shaped crystals were obtained in 1–2 days from the Wizard 4 screen condition #22 (Emerald biosystems, 25%(w/v) PEG 1500 PCB Buffer 7.0) using 0.5 µL of protein and 0.5 µL of crystallization solution equilibrated against 100 µL of the latter. Single crystals were transferred to a drop containing 80% crystallization solution and 20% 2-methyl-2,4-pentanediol (MPD) before freezing in liquid nitrogen for data collection. This structure was isomorphous to the previously determined wild-type ADH structure and contained 4 molecules in the asymmetric unit. An X-ray fluorescence scan indicated that cobalt was indeed present in the crystals. However, since the cobalt-complex was prepared from wild-type protein that contained Zn^2+^ ions, an X-ray fluorescence scan for Zn^2+^ was conducted as well which indicated that Zn^2+^ ions were also present. Structure solution was carried out by molecular replacement with Phase [26] via the Phenix [20] interface using ADH-WT as the search model. X-ray diffraction data for cobalt- bound PyAeADHII were collected at the following wavelengths using the same crystal: 1.60497 Å (cobalt-peak), 1.61018 Å (cobalt-low energy remote) and 1.28255 Å (Zn^2+^-peak). Phased anomalous difference maps were calculated using FFT [27] though the CCP4 [28] interface. Anomalous difference peaks were observed in the active sites using the cobalt-peak data. However, no peaks were observed using the cobalt-low energy remote data confirming that cobalt was present in the active sites. Likewise, anomalous difference peaks were present in the metal binding site using the Zn^2+^-peak data. Therefore, both Zn^2+^ and cobalt ions were modeled at these sites with 0.5 occupancies in the final structure (PDB: 4JBH).

### Crystallization and Data Collection of PyAeADHII bound to NADPH (ADH-NADPH)

The wild type ADH construct described above was incubated with 5 mM NADPH for 30 minutes in ice prior to screening. Plate shaped crystals were obtained in 1–2 days from the Wizard 3 screen condition #12 (Emerald biosystems, 10% (w/v) PEG 8000, 100 mM Hepes pH 7.5, 8%(v/v) ethylene glycol). Single crystals were transferred to a drop containing 75% crystallization solution and 25% ethylene glycol before freezing in liquid nitrogen for data collection. The Matthew's coefficient [29] was calculated based on an estimated molecular weight of 35,481 Da and indicated that there were most likely 16 molecules in the asymmetric unit (Vm = 2.8, 55.4% solvent; **Figure S1**). An initial solution was obtained by molecular replacement, using the coordinates from ADH-WT structure as the search model, with Phaser [26] within the Phenix [20] software package. Following refinement, the *R*-factor converged at approximately 35%. Examination of the resulting electron density maps revealed that certain residues near the NADPH binding pocket were in an incorrect conformation relative to the apo protein used for the search model. The model was then improved using the autobuilding suite within Phenix and a single subunit from the resulting model was used for subsequent molecular replacement searches with Molrep [30] to position the final four molecules. Following refinement of the 16 molecules, positive difference electron density (Fo-Fc) greater than 3σ was observed in the active sites of each subunit and was consistent with NADPH (**Figure S2**). However, it could not be determined if the ligand existed in the NADP^+^ form and was therefore modeled as NADPH. After fitting the NADPH molecules, the final structure was improved with subsequent rounds of refinement and manual model building (PDB: 4JBI).

### Structure Comparison with apo ADH and modeling of α-tetralone

For comparison, chain A of apo PyAeADHII was superimposed onto chain A of the NADPH bound structure from residue Met 1 to Pro 331 (328 residues, chain A) using Superpose [31].

### Modeling of α-tetralone in the active site of PyAEADHII

The binding model of substrate α-tetralone in the active site of PyAEADHII was predicted using a protocol with molecule docking and MD simulations, as reported previously [32]. Briefly, α-tetralone was docked into the active site of PyAeADHII/NADPH using AutoDock 4.2 [33]. The Lamarckian Genetic Algorithm (LGA) was applied to search the entire binding site of the protein which was defined by a grid of 70×70×70 points with a grid spacing of 0.5 Å centered at the co-factor NADPH. A total of 100 runs were generated and the maximum number of energy evaluations was set to 2×10^6^. Clustering analysis of the docked poses was performed using AutoDockTools [33] to identify the major binding modes based on the root-mean standard deviations (RMSD). To optimize the binding models, MD simulations were conducted for the ADH/NADPH/α-tetralone binding complex in explicit solvent using the AMBER 11 package with the ff99SB force field [34]. The solvated protein systems were first subjected to a gradual temperature increase from 0 K to 300 K over 100 ps, and then equilibrated for 500 ps at 300 K, followed by production runs of 2 ns length in total. Constant temperature and pressure (300 K/1 atm) were maintained during the time course of simulations with a time constant for heat-bath coupling of 0.2 ps. Trajectory analysis and the binding free energy calculations were performed using the PTRAJ and MMPBSA modules in the AMBER 11 package.

## Results and Discussion

### Miniaturized PyAEADHII assay for quantitative high-throughput screening (qHTS)

Working with hyperthermophilic enzymes is technically challenging in a typical laboratory environment due to the high temperatures required to yield the highest specific activity for these enzymes. Our previous work identified α-tetralone as a potential substrate for PyAeADHII, with α-tetralone converted to α-tetralol and oxidation of the coenzyme NADPH to form NADP^+^. For low-throughput experiments, we monitor NADPH oxidation as a decrease in A_340_ using a spectrophotometer. However, to make the assay more amenable to high-throughput formats, we measured the fluorescence of NADPH (λ_ex_  = 340 nm_,_ λ_em_  = 450 nm) using qHTS. In this mode the fluorescence signal in the assay decreases as NADPH is converted to NADP^+^.

Designing a high-throughput assay to screen for inhibitors of a hyperthermophilic enzyme like PyAEADHII is additionally challenging for a number of reasons. The first such challenge is that PyAEADH11 is catalytically most active at temperatures higher than 50°C [9] – temperatures that are difficult to maintain during the course of the reaction using the equipment necessary for HTS. For this reason, significant assay optimization was required to determine the concentration of enzyme that could be used in the assay at 25°C or 37°C –temperatures more amenable for use with HTS – such that sufficient substrate/coenzyme conversion took place within a certain time-course (see **Materials and Methods** for more details). Another challenge specific to working with this enzyme is that PyAeADHII shows very low activity for α-tetralone, the only known substrate for PyAeADHII, even at optimal temperatures [9], with µM levels of enzyme and mM concentrations of substrate required to measure sufficient activity. In spite of these significant challenges, however, we were able to identify a number of compounds that inhibited PyAeADHII using this high-throughput assay.

Another factor we considered when developing the assay was the effect of compound fluorescence on the assay results. Small molecule libraries, which contain a relatively high percentage of heterocyclic compounds with low levels of conjugation, are known to contain a significant fraction of compounds with blue fluorescence (∼5% of these libraries) which can interfere with fluorescence-based assay results when shorter excitation and emission wavelengths are used (λ_em_ ∼350****nm; λ_ex_ = 450–490****nm) [35], [36]. For this reason we incorporated a step in the protocol in which the fluorescent signal from the plate was read post-compound addition, but prior to initiation of the reaction. The final 1,536-well protocol for library screening is described in the **Table S1**.

**Figure 1:**
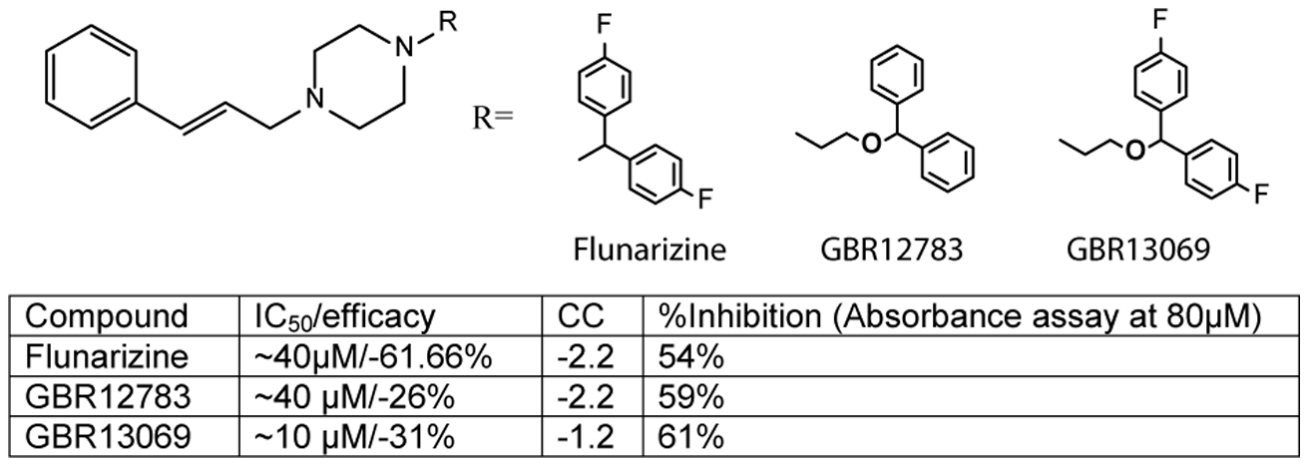
PyeADHII inhibitors identified in the HTS. Top, structure of inhibitors, bottom table shows the potency data. CC  =  qHTS curve class. None of the compounds showed autofluorescence.

**Figure 2:**
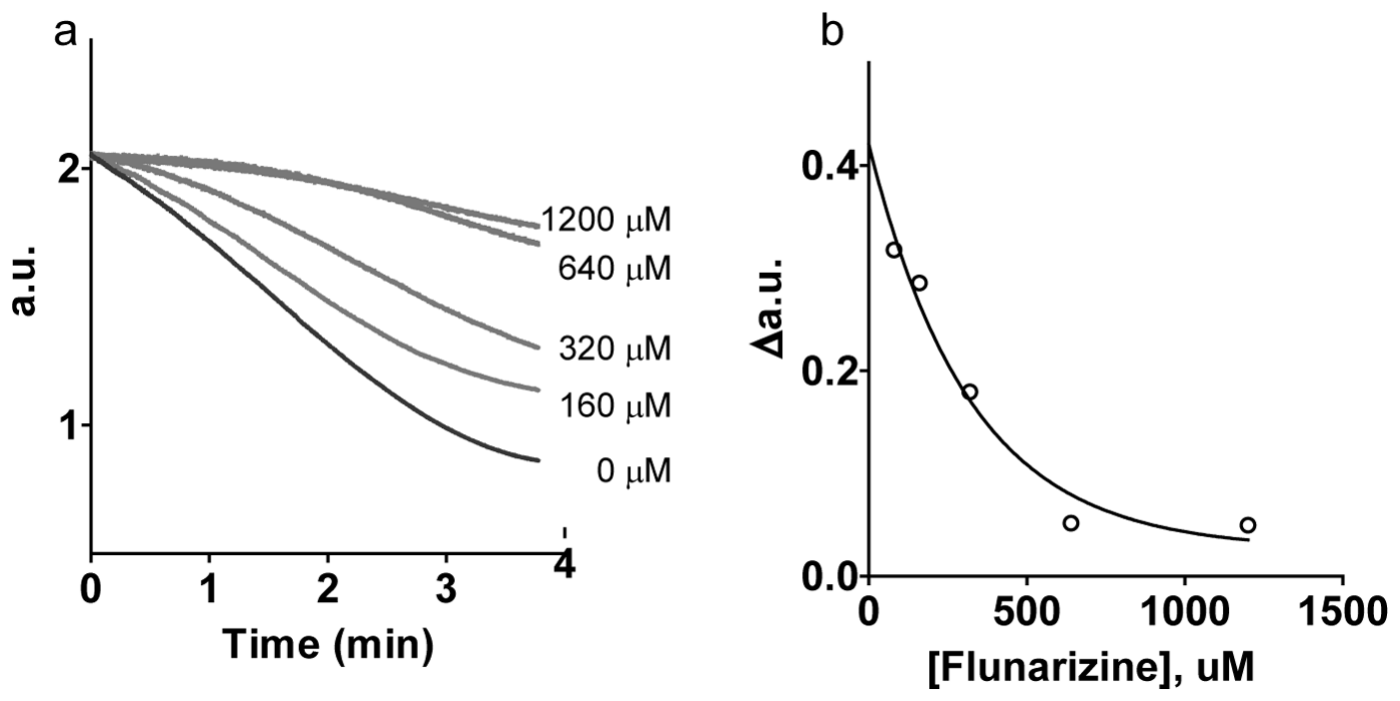
Inhibition of PyAeADHII at 70°C by flunarizine dihydrochloride. The assay was performed with 260 nM PyAeADHII in 50 mM NaPO4 pH 7.5, 3 mM α-tetralone, 0.3 mM NADPH in the presence of 80–1200 µM flunarizine dihydrochloride in DMSO/isopropanol. a) Absorbance of NADPH (340****nm) was measured over time at different flunarizine concentrations. b) The rate of NADPH conversion (Δa.u.  =  absorbance unit change/minute) versus flunarizine concentration.

We used this 1,536-well protocol to screen two libraries containing a total of approximately 2,700 compounds at seven concentration points. These libraries contained a variety of scaffolds, some of which contained alcohol and ketone groups, which could serve as more optimal ADH substrates, in addition to possibly acting as inhibitors or activators of the enzyme. For each assay plate we calculated the signal-to-background ratio (SB) and the Z' factor [37]. The screen performed well with minimal well-to-well variation (5%), a SB of approximately 3-5-fold, and Z' factors of between 0.5 and 0.9. From the screen, we were able confirm six compounds of a similar chemotype that showed an inhibitory effect on PyAeADHII, with IC_50_s ranging from 10 to 60 µM, and efficacies, for most, of approximately 30% (**Figure 1**). One compound, flunarizine dihydrochloride, had an efficacy of 60%. The activity of these compounds was confirmed using the spectrophotometry assay at 37°C. Inhibition of PyAeADHII by flunarizine dihydrochloride was also demonstrated in a more relevant assay conducted at 70°C (50–70% inhibition; **Figure 2**) with a significantly lower concentration of enzyme (260 nM). Thus the HTS assay developed was able to identify the first compounds known to inhibit hyperthermophilic PyAeADHII.

### Cobalt(II) substituted PyAeADHII

The sequence alignment obtained by comparing PyAeADHII, *Sulfolobus solfataricus* ADH (SsADH), *Saccharomyces cerevisiae* ADH (YADH) and horse liver ADH (HLADH) sequences [9], revealed that the structural Zn^2+^-binding site was conserved in PyAeADHII and that this enzyme lacked the key residues involved in coordinating to the catalytic Zn^2+^. To obtain experimental evidence for the coordination environment of the zinc sites in PyAeADHII in solution, we replaced the spectroscopically silent Zn^2+^ with cobalt [38]. Standard methods for cobalt substitution of the catalytic Zn^2+^ site involve the removal and the replacement of Zn^2+^from purified enzymes using chelating agents such as dipicolinic acid followed by dialysis, addition of cobalt, and dialysis again. In some cases, at the appropriate pH, direct metal exchange of the catalytic Zn^2+^ site can also be achieved [39], [40]. Metal substitution of the structural Zn^2+^ site is much more difficult to achieve. To address this, a method for direct cobalt incorporation in the Zn^2+^ binding sites of metal-enzymes by bacterial growth in the presence of Zn^2+^-depleted and cobalt-enriched media, was performed (see **Materials and Methods**).

**Figure 3:**
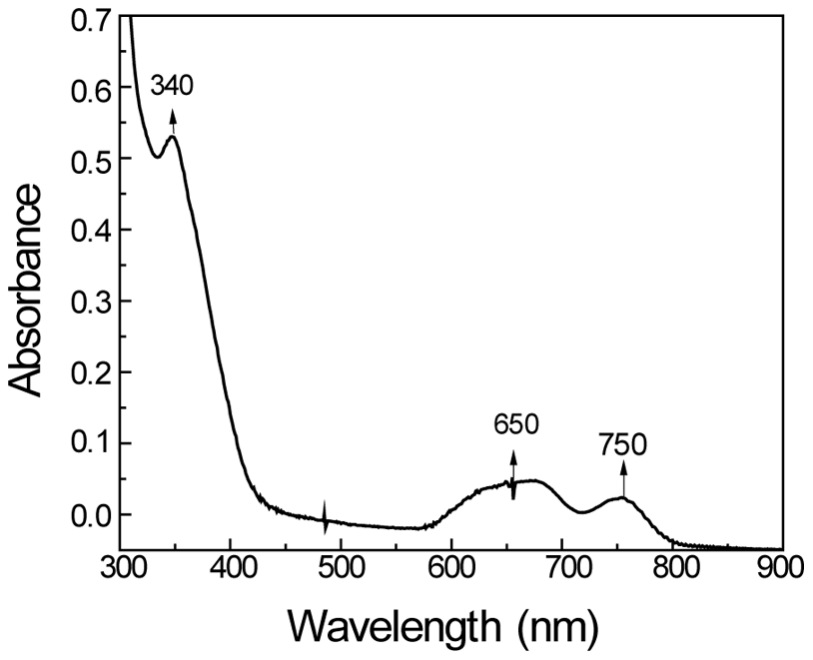
Absorption spectra of PyAeADHII Co-complex. The lack of a weak band at 520 nm and the presence of the 740 nm band indicates that cobalt was substituted at the structural site.

The absorption spectrum of the putative ADH-cobalt complex is shown in **Figure 3**. The spectrum shows the characteristic ligand-metal charge transfer band at 340 nm in addition to absorbance peaks in the visible region. In HLADH, the visible electronic absorption spectrum of the cobalt-substituted enzyme that contains cobalt only at the catalytic site, is characterized by a major absorbance peak at 640 nm and two minor peaks at 530 nm and 550 nm [38]. However, when the Zn^2+^ ion is replaced by cobalt at the structural site, the spectra shows a characteristic absorption peak at 740 nm in addition to the 640 nm peak [38]. The spectra recorded by the cobalt-substituted PyAeADHII gave broad peaks at 740–750 nm and 640 nm, characteristic of cobalt insertion at the structural site, but not in the 530–550 nm region (which is characteristic of the cobalt substitution at the catalytic site). However, due to the fact that the known absorbance peaks found for the catalytic site in HLADH (530–550 nm region) are typically weak, it was difficult to determine if cobalt was also substituted at a putative catalytic site. Therefore, although this experiment was able to conclusively demonstrate that cobalt was substituted at the structural Zn^2+^ site, and confirm that wild-type PyAeADHII thus contains structural Zn^2+^, it could not definitively conclude whether or not the enzyme contained an additional Zn^2+^ site.

### Crystal Structure and X-ray fluorescence of wild-type and cobalt-substituted PyAeADHII

**Figure 4:**
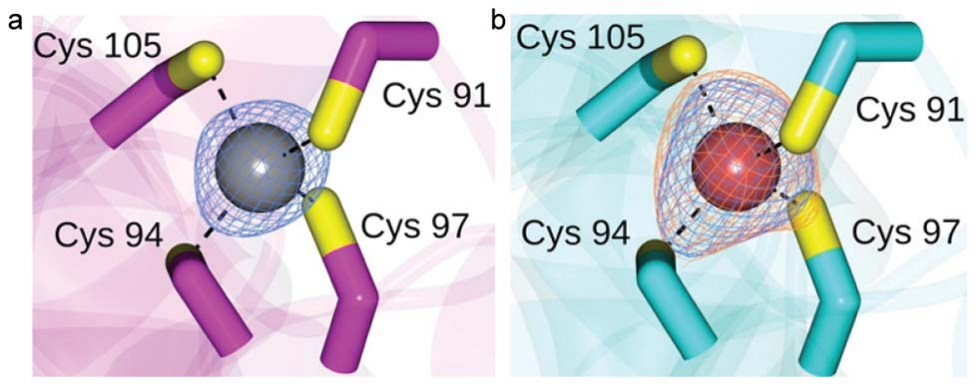
The Zn^2+^ binding site of PyAeADHII. **a) Zn^2+^ binding in the active site of ADH-WT subunit A.** The phased anomalous map contoured at 5σ calculated using the Zn-peak data, is shown as blue mesh. b) Metal binding site for Co-substituted PyAeADHII with the cobalt ion drawn as a red sphere. Phased anomalous difference maps contoured at 5σ were calculated using Co-peak data (orange) and Zn^2+^-peak data (blue) and revealed that both ions were present.

We determined the X-ray structure of wild-type PyAeADHII at 1.75 Å (crystallographic data summarized in **Table S2**). Four molecules of ADH were found in the asymmetric unit with each monomer containing its own structural Zn^2+^ (**Figure S3**). To confirm that the metal-binding sites in the wild-type enzyme were complexed to Zn^2+^, a low energy remote data set was collected using the same crystal at λ = 1.2480 Å. No peaks were observed in the anomalous difference map when using data collected at this wavelength, indicating that the metal assignment was correct (**Figure 4a**). This result thus confirms the hypothesis (generated from sequence alignment analysis and the absorption spectra of the cobalt substituted enzyme) that only the structural Zn^2+^site is present in PyAeADHII. The Cys-Zn^2+^ distances are: Cys-91  = 2.31Å, Cys-94  = 2.29Å, Cys-97  = 2.32Å and Cys-105  = 2.32Å (**Figure 4a**). Therefore, this structural site shows Cys-Zn^2+^ ligand distances which are akin to peptide-zinc model systems, as opposed to what has been observed in some ADH enzymes where longer Cys-Zn^2^ distances have been observed (2.69 Å) suggesting an entatic state for this site in these enzymes [41].

We also determined the X-ray structure of the cobalt substituted PyAeADHII at 2.20 Å (**Table S2**). Anomalous difference peaks were observed in the active sites using the cobalt-peak data. However, no peaks were observed using the cobalt-low energy remote data, confirming that cobalt was present in the structural metal binding site. Likewise, anomalous difference peaks were present in the structural metal binding site using the Zn^2+^-peak data. Therefore, both Zn^2+^ and cobalt ions were modeled at these sites with 0.5 occupancies. Anomalous difference maps for the metal binding site in subunit A is shown in **Figure 4b**. These data indicate that the incorporation of cobalt within the structural sites was partial, and the presence of cobalt did not change the folding of the enzyme.

### Comparison of PyAeADHII structure to other ADH enzymes

**Figure 5:**
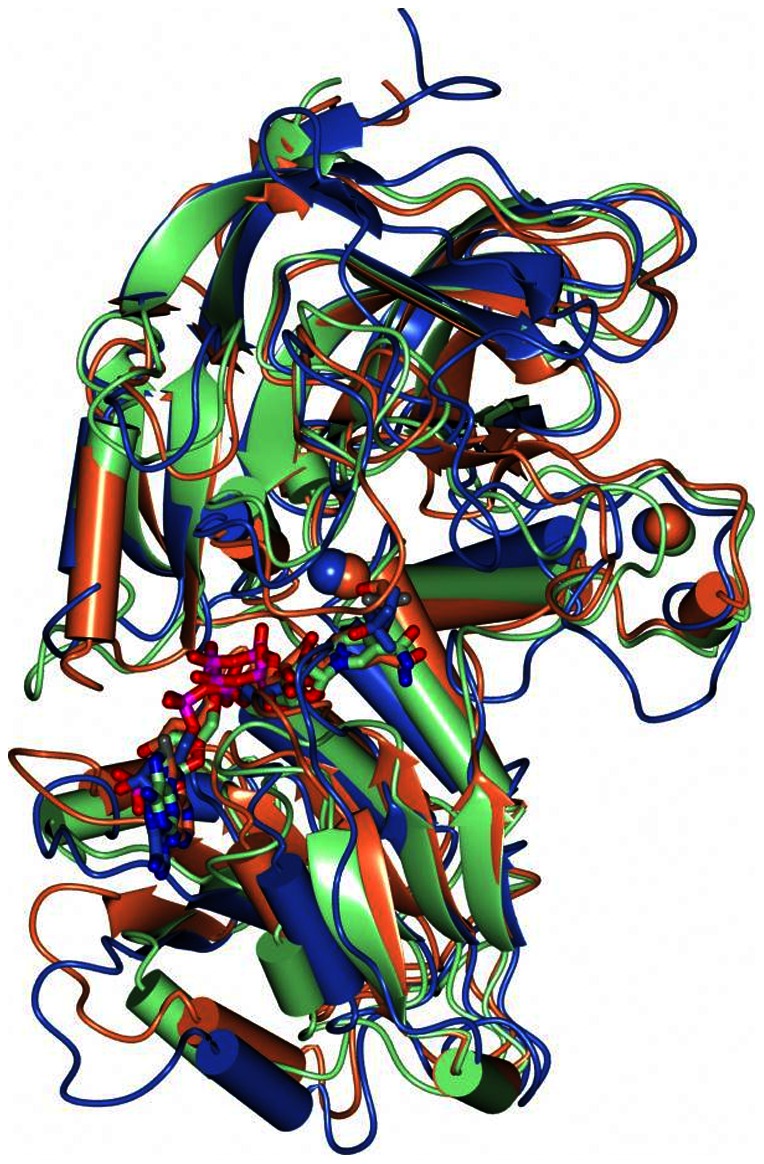
Superposition of ADH-NADPH (green) with YADH (coral, PDB 2HCY) and HADH (blue, PDB 5ADH). Active site ligands are drawn as cylinders and Zn^2+^ ions are represented as spheres.

To examine the structural overlap with higher order organisms, the three-dimensional structure of PyAEADHII was compared with horse liver (HADH, PDB: 5ADH), and yeast (YADH, PDB: 2HCY) alcohol dehydrogenases which indicated that a similar fold is adopted (**Figure 5**). The superposition of HADH and YADH with ADH-NADPH was conducted using secondary structure matching [31] at 1.73 Å (309 residues) and 1.92 Å (319 residues), respectively. Consistent with other studies which have compared mesophilic and thermophiliic homologues [9], the arrangement of secondary and tertiary structures doesn't explain the large difference in stability.

It has been suggested that increased thermostability of certain alcohol dehydrogenases may likely be attributed to the increased number of proline residues located at specific sites such as coil/loop regions which serve to stabilize the global structure [42]. It was noted, for example, that ADH from the thermophilic bacteria *Thermoanaerobacter brockii* (TBADH) and HADH contain a high number of proline residues (21 and 19 respectively) relative to YADH which contains 13 proline residues [43]. PyAeADHII contains 19 proline residues of which 14 reside in coil/loop regions of the protein. A comparison of the proline residue distribution for PyAeADHII, TBADH (PDB: 1YKFl, [44]), HADH and YADH is shown in **Figure S4**. Notably, the proline residues in PyAeADHII, TBADH and HADH are distributed throughout the protein whereas in YADH, the prolines are localized in the N-terminal and central regions of the protein. This distribution of proline residues in PyAeADHII may therefore contribute to its observed thermostability.

### Co-crystal structure of PyAeADHII with NADPH and analysis of the putative active site

**Figure 6:**
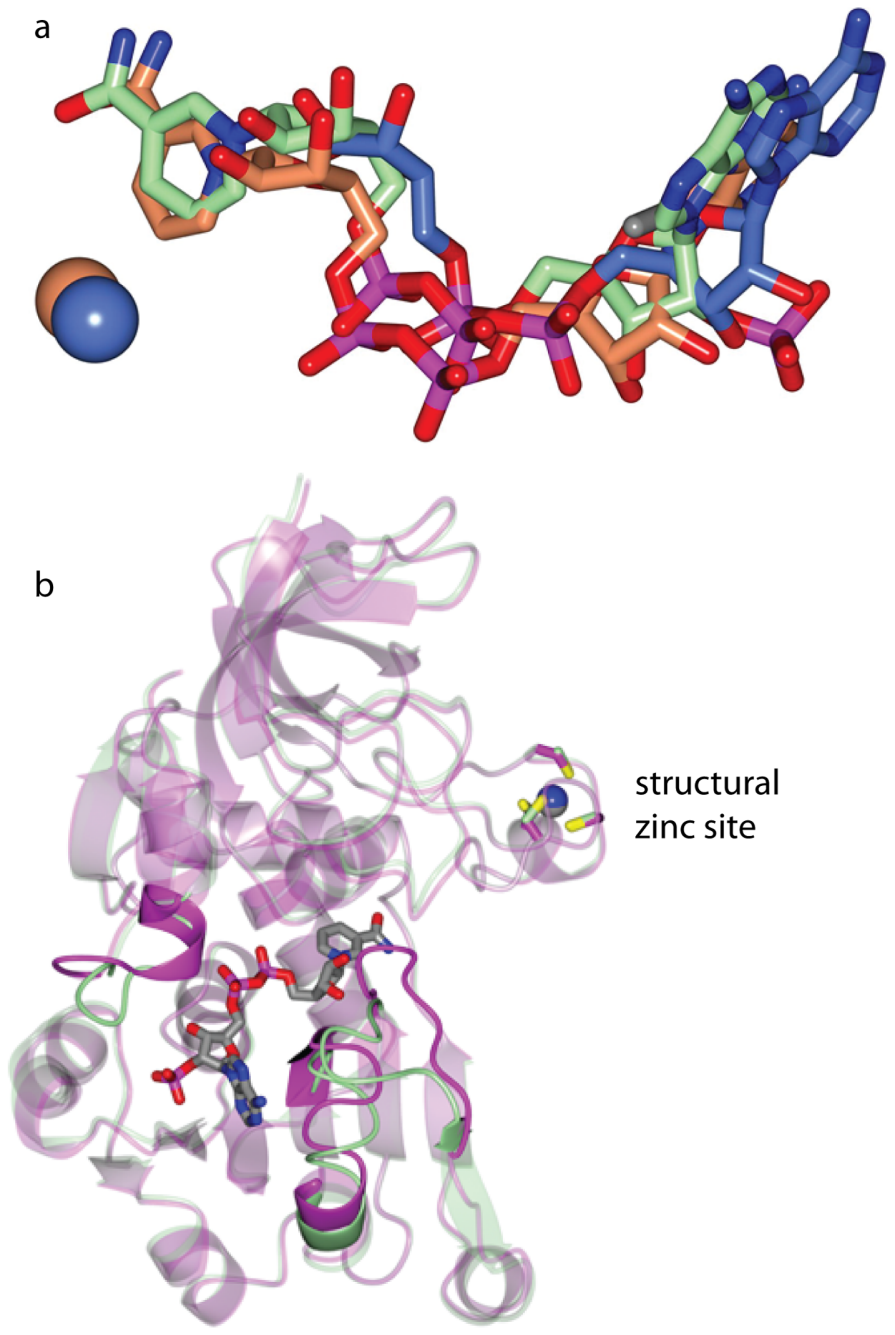
Active site view of ADH-NADPH (green) with YADH (coral, PDB 2HCY) and HADH (blue, PDB 5ADH) (a). YADH and HADH have adenosine-5-diphosphoribose and nicotinamide-8-iodo-adenine-dinucleotide bound in the active site respectively. Active site ligands are drawn as cylinders and active site Zn^2+^ ions for YADH and HADH are represented as spheres. b) Superposition of ADH-WT (magenta) and ADH-NADPH (green). The NADPH molecule and Cys residues in the metal binding site are drawn as cylinders. The Zn^2+^ ions associated with ADH-WT and ADH-NADPH are drawn as grey and blue spheres respectively. Regions with the largest conformational differences are highlighted.

**Figure 7:**
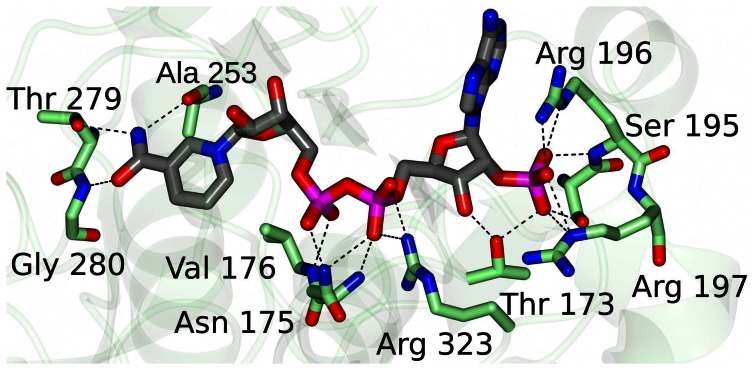
Residues of PyAeADHII that form hydrogen bonds with NADPH. Hydrogen bond interactions are shown as dashed lines between NADPH and active site residues (green) for the ADH-NADPH structure.

To better define the active site of PyAeADHII, we determined the co-crystal structure of PyAeADHII monomer bound to NADPH (**Figure S1**; PDB: 4JBI). This co-crystal structure revealed a similar binding mode for NADPH in PyAeADHII as is found for other ligands in the HADH and YADH enzymes (**Figure 6a**). The NADPH bound structure is similar to apo PyAeADHII with an overall RMSD of 1.02 Å between Cα atoms (**Figure 6b**). However, certain regions near the NADPH binding site undergo conformational changes to accommodate ligand binding. Specifically, the residues between Asp-230 to Ser-240, Ala-253 to Val-261 and Glu-316 to Val-324 are in different conformation in the NADPH bound structure (**Figure S5** and **S6**). Both Arg-323 and 326 conform to bind to the phosphates of NADPH and the main-chain NH of Ala-253 form H-bonds with the nicotinamide carbonyl which involves movement of a loop region (see **Figure 7**, **Figure S6**, and **Movie S1**). Similarly, the active site ligands for HADH and YADH adopt various hydrogen bonds to the protein residues (**Figure S7a and b**). The main difference, apart from the distinct amino acid residues that interact with the ligands for each structure, is the lack of a Zn^2+^ ion in the active site for PyAeADHII – NADPH as is present for HADH and YADH (**Figure S7c**). The active site Zn^2+^ is coordinated by two Cys residues and a His residue for HADH and YADH. However, ADH-NADPH does not contain the requisite amino acid residues in this region for metal coordination but instead Asn-39, Ser-61 and Ile-147 occupy this region. The Cys ligands in the structural Zn^2+^ site show little change between apo and NADPH bound structures (**Figure S8**).

### Modeling of α-tetralone into the NADPH-bound structure

**Figure 8:**
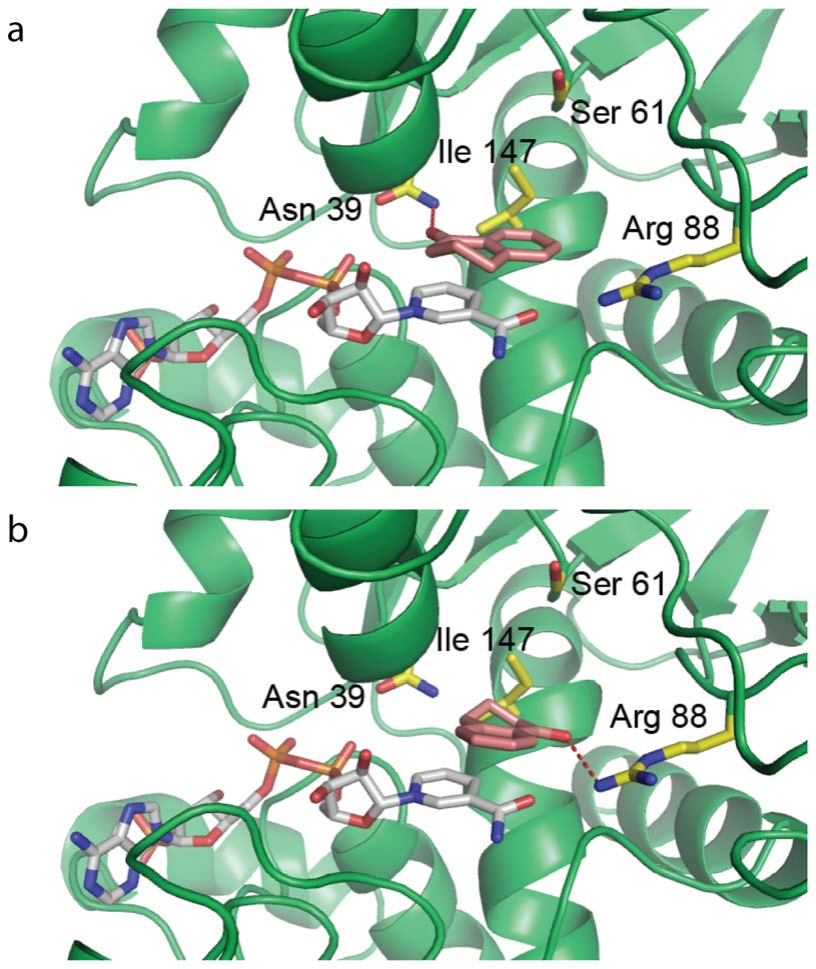
Binding interaction of α-tetralone in the substrate binding site of PyAeADHII/NADPH predicted by AutoDock. a) Binding mode 1, α-tetralone was positioned on top of the nicotinamide ring as a stacking interaction, and the oxygen atom formed a H-bonding interaction with the side chain of residue Asn-39 (3.5 Å). b) α-tetralone again forms stacking interaction with nicotinamide ring, but the oxygen atom was orientated towards residue Arg-88 forming a H-bonding interaction (3.0 Å). PyAeADHII is shown as a cartoon (green) and residues in the substrate binding site are shown as sticks (carbon colored in yellow, nitrogen in blue, oxygen in red). NADPH (carbons are in grey) and the substrate α-tetralone (carbon are in orange) are shown as sticks.

To possibly identify residues in the active site of PyAeADHII we modeled the substrate α-tetralone into the NADPH-bound structure. Two plausible binding modes of the substrate in the active site were predicted from docking analysis (**Figure 8**). In the first binding mode, α-tetralone was positioned on top of the nicotinamide of NADPH forming π-stacking interaction, while the carbonyl oxygen atom pointed towards residues Asn39 in the deep binding pocket and formed a hydrogen-bonding interaction (**Figure 8a**). In the second binding model the substrate was bound in the active site of PyAeADHII in a similar manner (being situated on top of the nicotinamide of NADPH), but the oxygen atom was orientated on the opposite side and formed hydrogen-bonds with residue Arg-88 (**Figure 8b**). Further MD simulations showed that α-tetralone in the two binding models remained stable and the calculated binding free energies were comparable. Without a catalytic zinc ion in the active site, α-tetralone is accommodated well in the binding pocket of PyAeADHII. The binding affinities for α-tetralone appear to be mainly due to an aromatic stacking interaction with the nicotinamide ring of NADPH and H-bonding with either residue Arg-88 or Asn-39 in the two binding models. Asn-39 is a cysteine involved with Zn^2+^- coordination in other ADHs and is unique to PyAeADHII. Arg-88 is unique to PyAeADH – in HADH, Thr-94 is involved with this H-bonding, and in YADH, this residue is Leu-93. It is possible that Arg-88 acts to stabilize the oxyanion in the active site and upon hydride transfer this residue could donate a proton to form the alcohol.

## Conclusion

We studied both the functional and structural details of a thermostable ADH from archaea *P. aerophilum* with a substrate specificity and catalytic mechanism different than other well characterized medium- chain Zn- dependent ADH enzymes which contain both structural and catalytic Zn^2+^ ions. Short-chain dehydrogenases/reductases (SDR) are a large family of NADP(H)-dependent oxidoreductases that also contain the classic Rossmann fold motif for nucleotide binding but do not contain Zn^2+^ [45]. PyAeADHII, possessing only the structural Zn^2+^ site, may be a hybrid between Zn^2+^-dependent medium-chain ADHs and non- Zn^2+^ dependent SDRs. The characterization of this unique ADH presented here expands our understanding of the class of ADH enzymes and could help the design of thermostable dehydrogenases/reductases with new catalytic mechanisms.

## Supporting Information

Figure S1 **Asymmetric unit of ADH-NADPH showing the 16 molecules colored by chain.** The Zn^2+^ ions are drawn as grey spheres and NADPH molecules are shown as cylinders.
(TIF)

Figure S2 **Fo-Fc omit map contoured at 3 σ for the NADPH molecule associated with chain A in the ADH-NADPH structure.**
(TIF)

Figure S3 **The four molecules of ADH in the asymmetric unit colored by subunit.** Zinc ions are represented at grey spheres.
(TIF)

Figure S4 **Comparison of the distribution of proline residues (red) in ADH-NADPH (green), HADH (blue, PDB: 5ADH), TBADH (gold, PDB: 1YKF) and YADH (coral, PDB: 2HCY).** The structural Zn^2+^ ions are represented as spheres except for TBADH which does not contain a Zn^2+^ ion in this region.
(TIF)

Figure S5 **Plot of RMSD deviations per residue between Cα atoms of ADH-WT and ADH-NADPH.**
(TIF)

Figure S6 **Superposition of ADH-NADPH (green) and ADH-WT (magenta) showing the conformational changes observed in regions near the NADPH binding site.** The NADPH molecule is drawn as cylinders.
(TIF)

Figure S7 **Hydrogen bond interactions between the active site ligands and protein for a) HADH and b) YADH.** C) Active site metal binding region for HADH (blue), YADH (coral) superimposed with ADH-NADPH (green). Zn^2+^ ions are drawn as spheres.
(TIF)

Figure S8 **Comparison of the Zn^2+^ binding site for ADH-WT (magenta) and ADH-NADPH (green).** The Zn^2+^ ions associated with ADH-WT and ADH-NADPH are drawn as grey and blue spheres respectively.
(TIF)

Movie S1 (WMV)

Table S1 **1536 well protocol of PyAeADHII assay at 37°C.**
(DOCX)

Table S2 **Crystallographic data for PyAeADHII.**
(DOCX)
