# Supplementary figures and images for: Physicochemical Characterization of a Thermostable Alcohol Dehydrogenase from Pyrobaculum aerophilum
Source: PLoS One. 2013 Jun 5;8(6):e63828. doi: 10.1371/journal.pone.0063828 (PMC3673990; doi:10.1371/journal.pone.0063828)

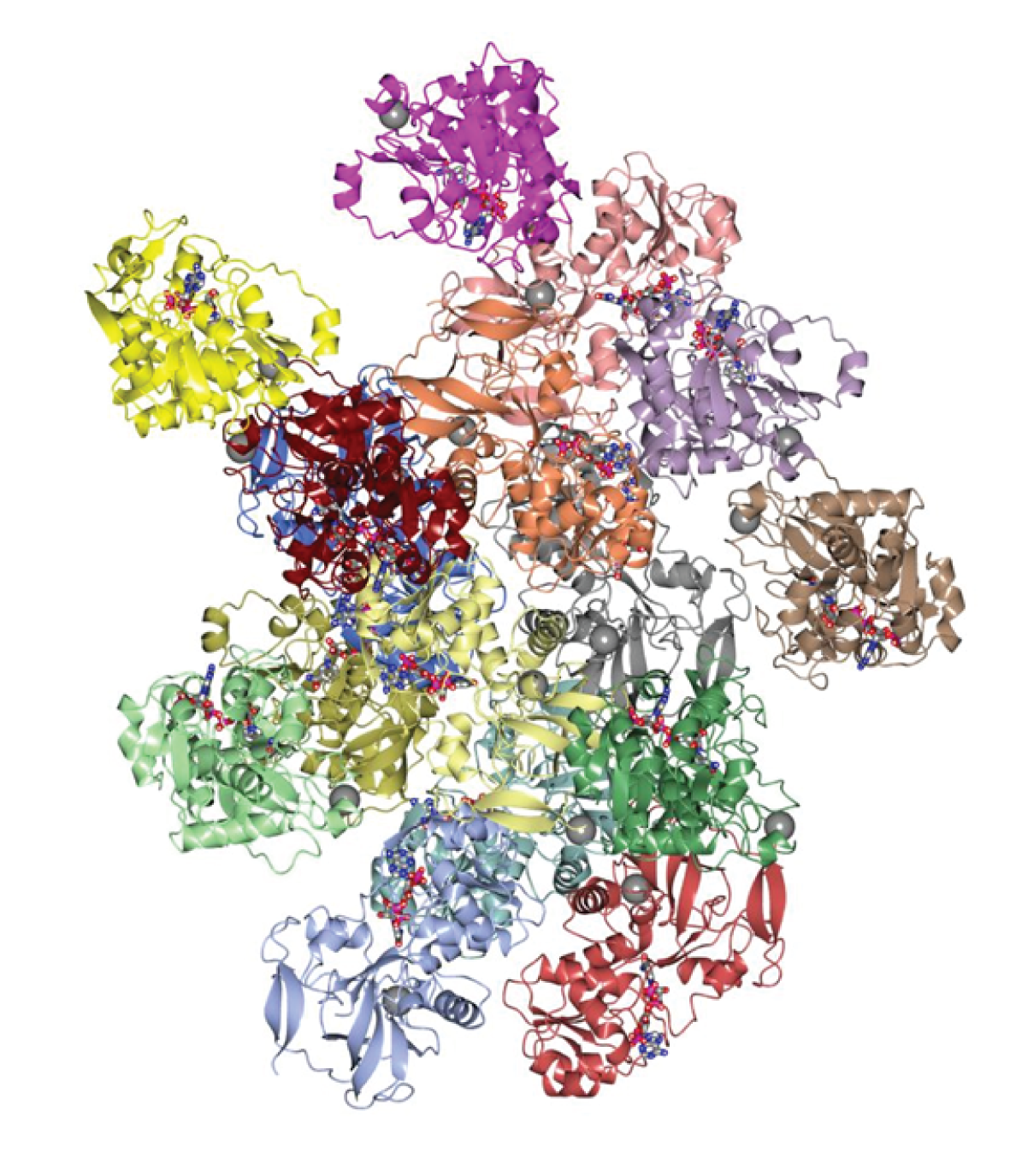

Supplement: Figure S1 — Asymmetric unit of ADH-NADPH showing the 16 molecules colored by chain. The Zn2+ ions are drawn as grey spheres and NADPH molecules are shown as cylinders. (TIF) [file pone.0063828.s001.tif]

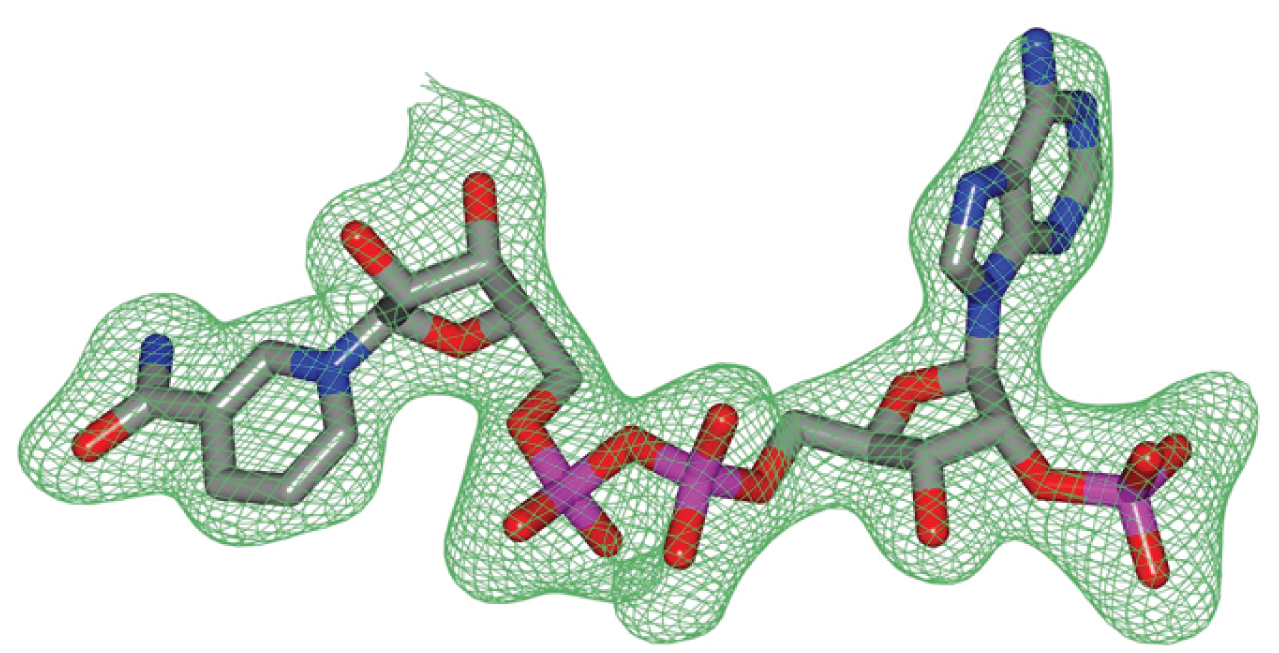

Supplement: Figure S2 — Fo-Fc omit map contoured at 3 σ for the NADPH molecule associated with chain A in the ADH-NADPH structure. (TIF) [file pone.0063828.s002.tif]

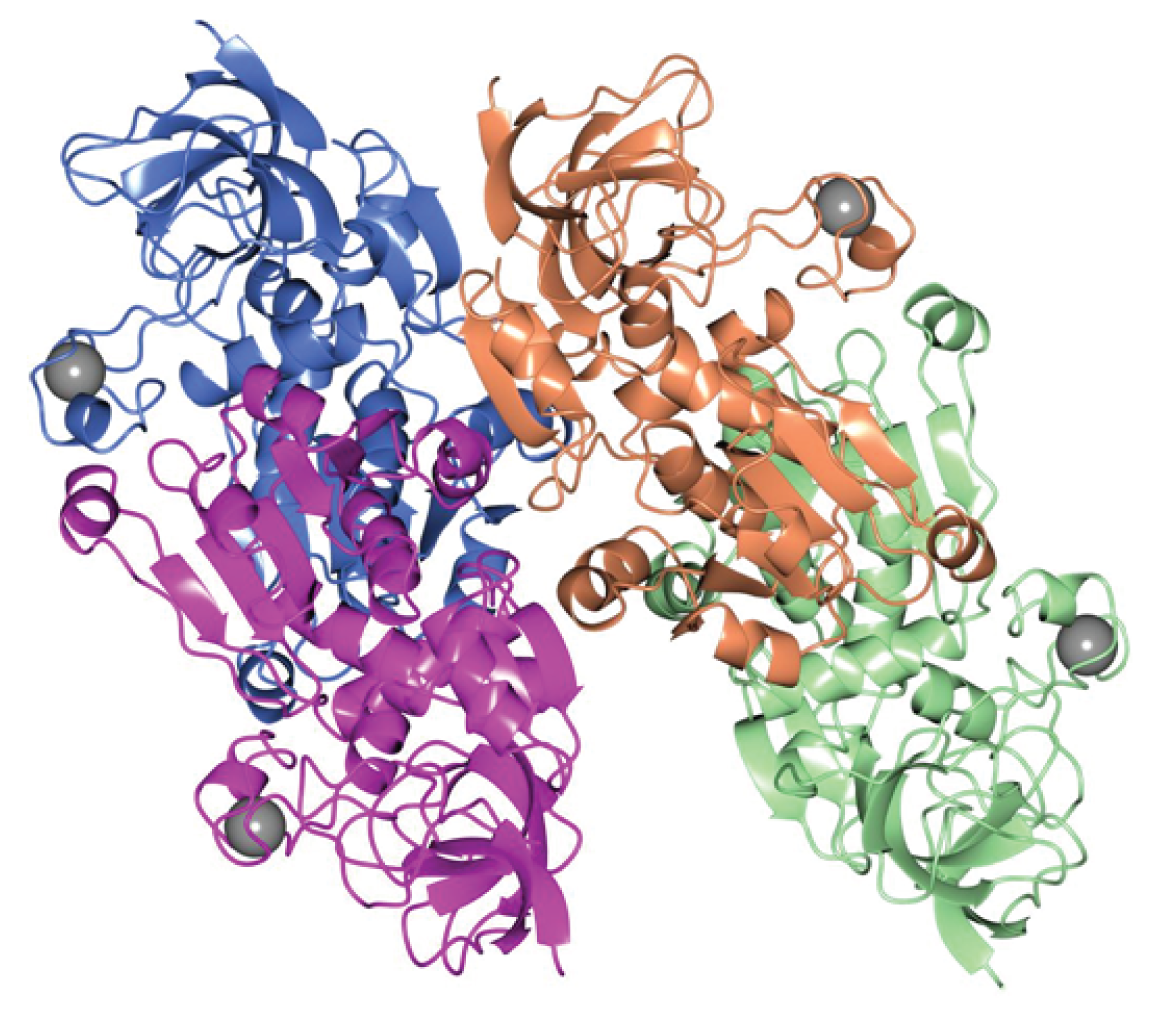

Supplement: Figure S3 — The four molecules of ADH in the asymmetric unit colored by subunit. Zinc ions are represented at grey spheres. (TIF) [file pone.0063828.s003.tif]

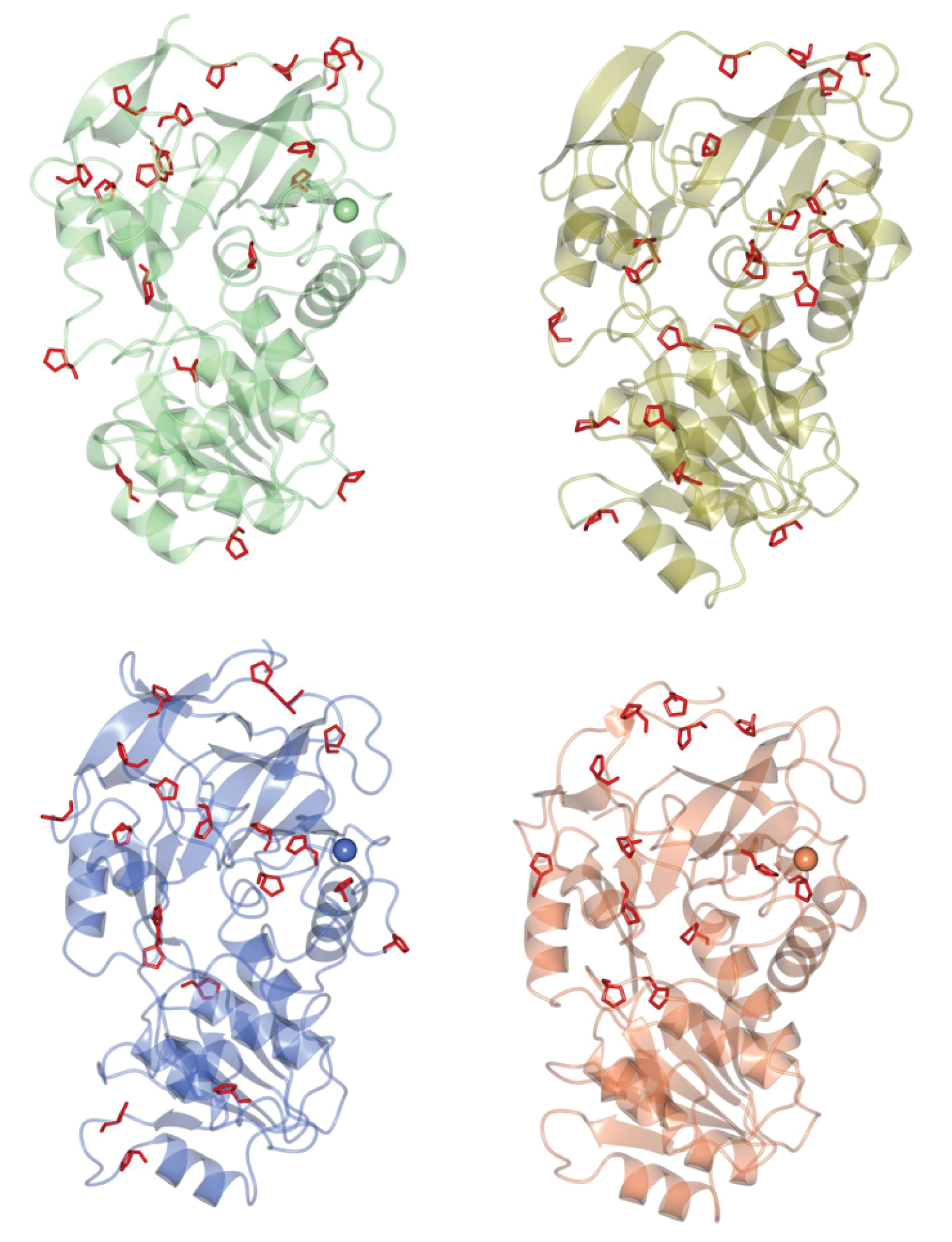

Supplement: Figure S4 — Comparison of the distribution of proline residues (red) in ADH-NADPH (green), HADH (blue, PDB: 5ADH), TBADH (gold, PDB: 1YKF) and YADH (coral, PDB: 2HCY). The structural Zn2+ ions are represented as spheres except for TBADH which does not contain a Zn2+ ion in this region. (TIF) [file pone.0063828.s004.tif]

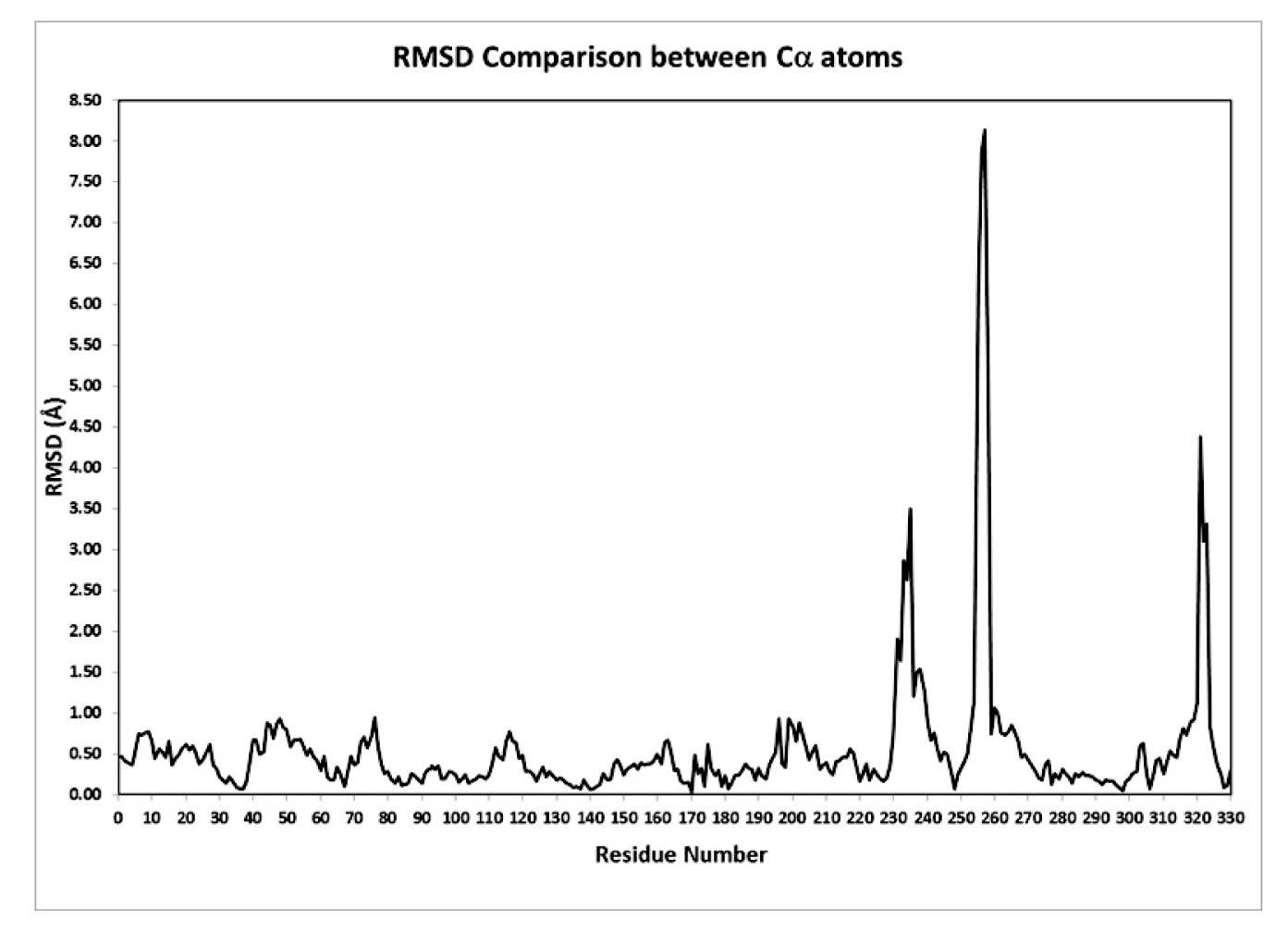

Supplement: Figure S5 — Plot of RMSD deviations per residue between Cα atoms of ADH-WT and ADH-NADPH. (TIF) [file pone.0063828.s005.tif]

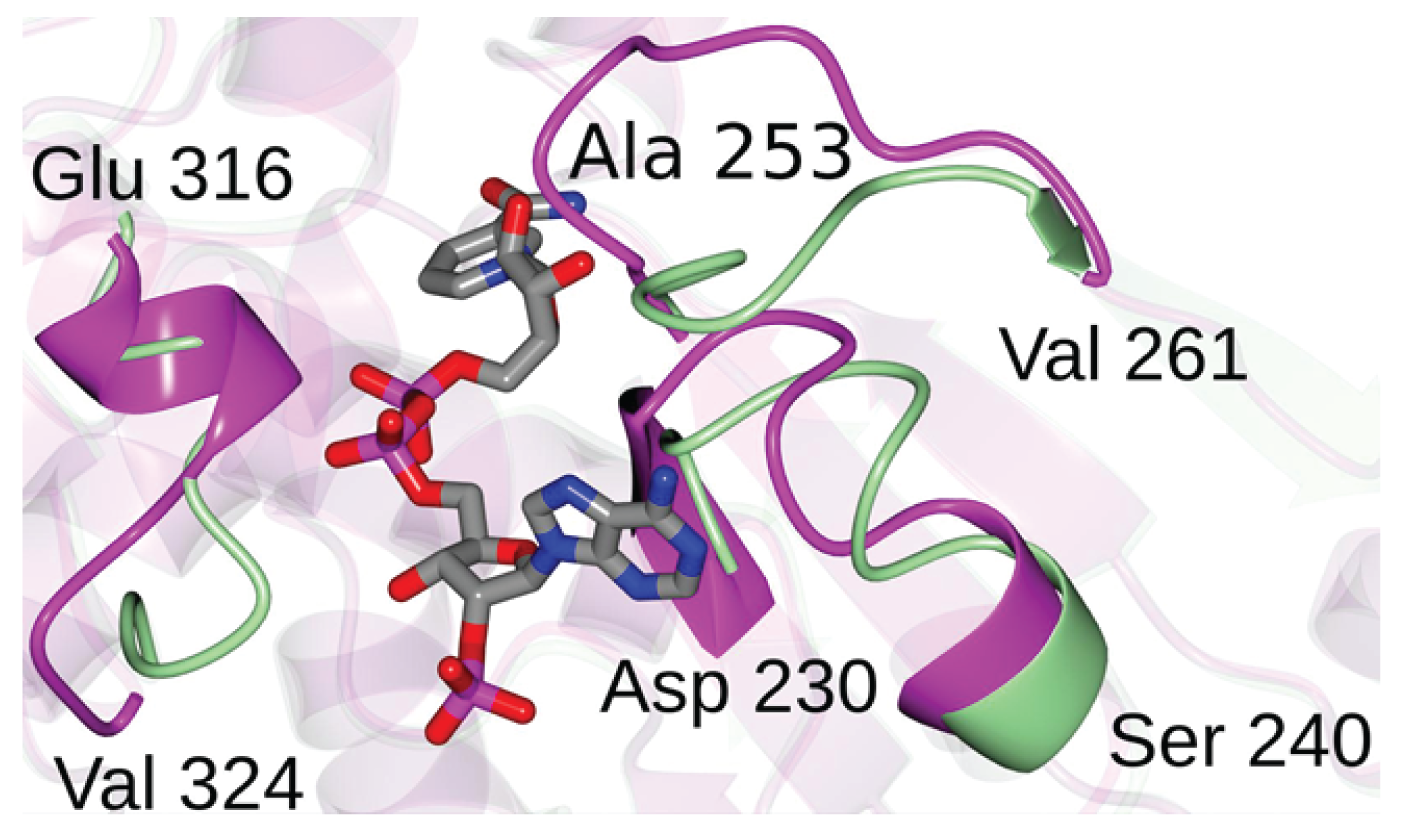

Supplement: Figure S6 — Superposition of ADH-NADPH (green) and ADH-WT (magenta) showing the conformational changes observed in regions near the NADPH binding site. The NADPH molecule is drawn as cylinders. (TIF) [file pone.0063828.s006.tif]

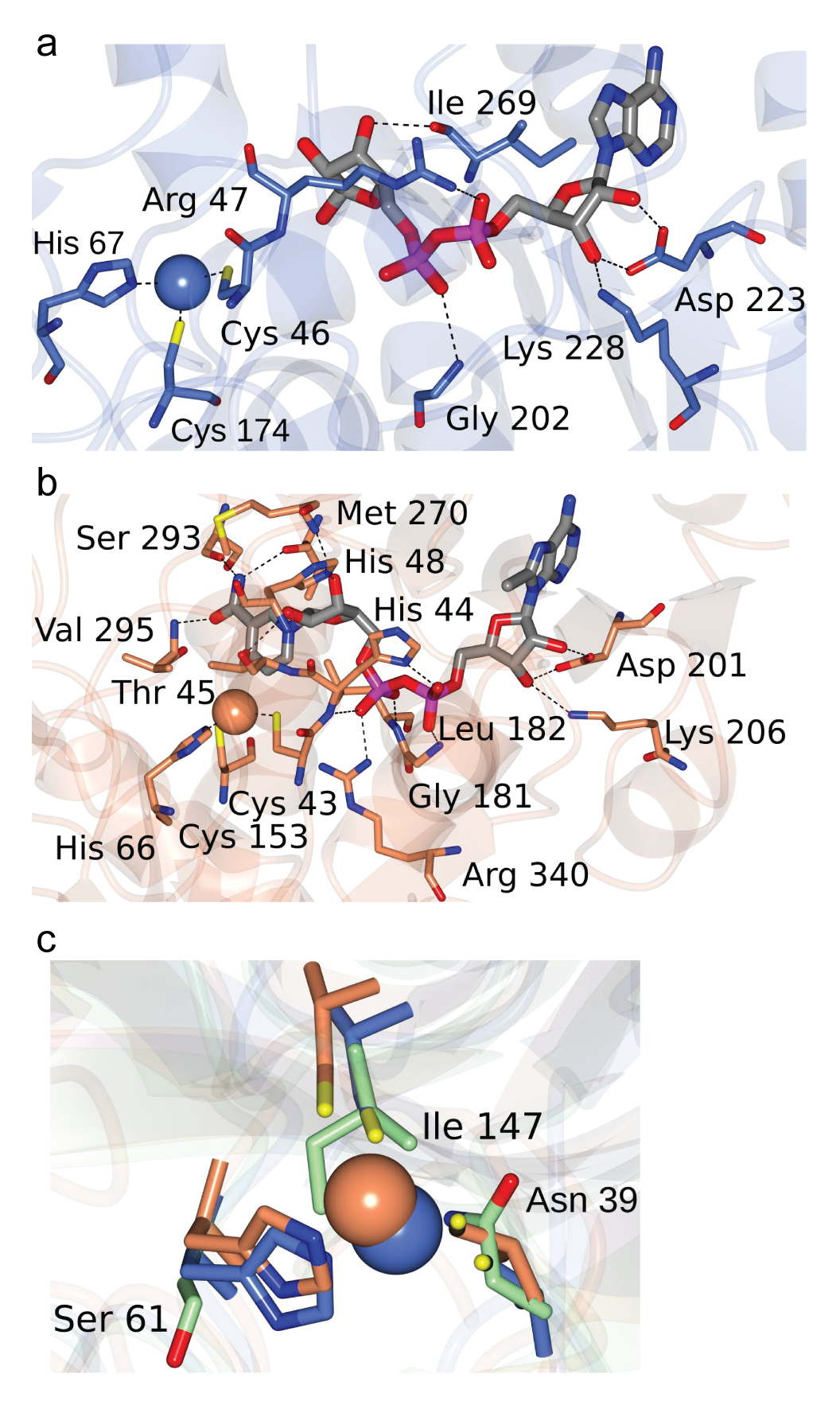

Supplement: Figure S7 — Hydrogen bond interactions between the active site ligands and protein for a) HADH and b) YADH. C) Active site metal binding region for HADH (blue), YADH (coral) superimposed with ADH-NADPH (green). Zn2+ ions are drawn as spheres. (TIF) [file pone.0063828.s007.tif]

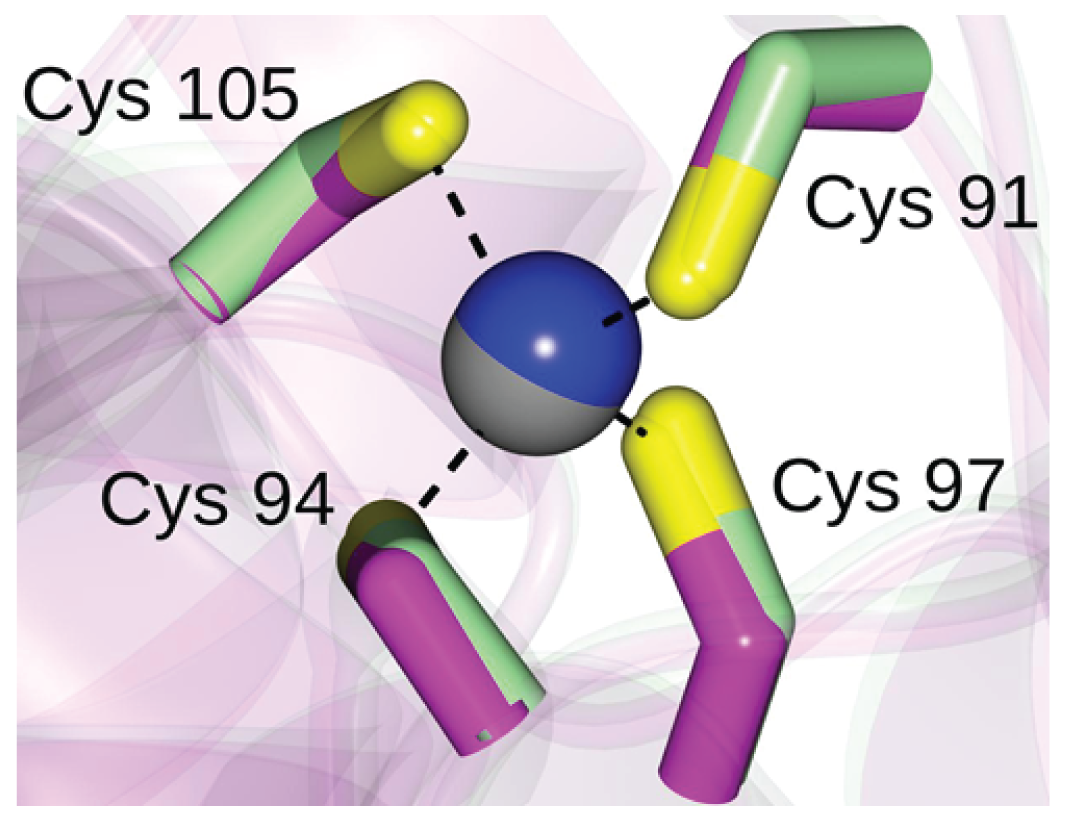

Supplement: Figure S8 — Comparison of the Zn2+ binding site for ADH-WT (magenta) and ADH-NADPH (green). The Zn2+ ions associated with ADH-WT and ADH-NADPH are drawn as grey and blue spheres respectively. (TIF) [file pone.0063828.s008.tif]
