# Supplementary material for: Physicochemical Characterization of a Thermostable Alcohol Dehydrogenase from Pyrobaculum aerophilum
Source: PLoS One. 2013 Jun 5;8(6):e63828. doi: 10.1371/journal.pone.0063828 (PMC3673990; doi:10.1371/journal.pone.0063828)
Supplement: Table S1 — 1536 well protocol of PyAeADHII assay at 37°C. (DOCX) [file pone.0063828.s010.docx]

**Table S1: 1536 well protocol of PyAeADHII assay at 37 °C**

| Sequence | Parameter | Value | Description |
| --- | --- | --- | --- |
| 1. | Reagent | 3µL | 2.6µM PyAeADHII |
| 2. | Compound | 23nL | Test compounds |
| 3. | Detection |  | Viewlux EX 340nm, EM 450nm |
| 4. | Reagent | 3µL | 0.3mM NADPH, 3mM alpha-tetralone |
| 5. | Time | 20min | Incubation at 37ºC |
| 6. | Detection |  | Viewlux |
| Sequence | Notes | | |
| 1 | enzyme dispensed in 1,536 -well plates, black solid bottom plate. | | |
| 2 | Compound addition with pin-tool | | |
| 3 | pre-read plate for fluorescent compounds | | |
| 4 | 0.3mM NADPH, 3mM alpha-tetralone | | |
| 5 | Incubation in the preheated oven at 37 °C for 10 min to warm plates and 10 min for reaction | | |
| 6 | EX 340nm, EM 450nm | | |
