# Supplementary material for: Physicochemical Characterization of a Thermostable Alcohol Dehydrogenase from Pyrobaculum aerophilum
Source: PLoS One. 2013 Jun 5;8(6):e63828. doi: 10.1371/journal.pone.0063828 (PMC3673990; doi:10.1371/journal.pone.0063828)
Supplement: Table S2 — Crystallographic data for PyAeADHII. (DOCX) [file pone.0063828.s011.docx]

**Table S2.** Crystallographic data for PyAeADHII

|  | **ADH-WT**  **(Zn SAD data)** | **ADH-WT**  **(PDB: 4JBG)** | **ADH-Co**  **(PDB: 4JBH)** | **ADH-NADPH**  **(PDB: 4JBI)** |
| --- | --- | --- | --- | --- |
| **Data Collection** |  |  |  |  |
| Unit-cell parameters  (Å, ^o^) | *a*=68.53  *b*=126.25  *c*=83.95  *β*=108.4 | *a*=68.51  *b*=126.40  *c*=84.06  *β*=108.5 | *a*=68.40  *b*=125.34  *c*=82.94  *β*=107.1 | *a*=138.13  *b*=169.26  *c*=149.15  *β*=116.3 |
| Space group | *P*2_1_ | *P*2_1_ | *P*2_1_ | *P*2_1_ |
| Resolution (Å)^1^ | 126.25 – 1.82  (1.92-1.82) | 50.0 - 1.75  (1.78-1.75) | 49.16-2.20  (2.25-2.20) | 50.00-2.35  (2.39-2.35) |
| Wavelength (Å) | 1.2819 | 1.0000 | 1.60497 | 1.0000 |
| Temperature (K) | 100 | 100 | 100 | 100 |
| Observed reflections | 669,534 | 897,961 | 434,572 | 892,813 |
| Unique reflections | 109,201 | 136,008 | 64,634 | 254,725 |
| <I/σ(I)>^1^ | 18.7 (2.9) | 13.3 (2.8) | 13.1 (3.6) | 8.5 (1.7) |
| Completeness (%)^1^ | 90.6 (55.9) | 99.8 (99.9) | 95.6 (73.5) | 99.9 (100) |
| Multiplicity^1^ | 6.1 (3.7) | 6.6 (6.7) | 6.7 (5.6) | 3.5 (3.6) |
| *R*_merge_ (%)^1, 2^ | 6.3 (50.2) | 8.6 (71.8) | 9.7 (51.0) | 13.2 (81.0) |
| *R*_meas_ (%)^1, 4^ | 7.7 (65.8) | 9.4 (77.8) | 10.5 (56.3) | 15.6 (95.5) |
| *R*_pim_ (%)^1, 4^ | 3.0 (33.5) | 3.6 (29.8) | 4.0 (23.3) | 8.2 (50.2) |
| CC_1/2_ ^1, 5^ |  | 0.998 (0.790) | 0.997 (0.790) | 0.991 (0.592) |
| **Phasing** |  |  |  |  |
| Estimated mean FOM | 0.700 | - | - | - |
| Pseudo-free CC (%) | 70.80 | - | - | - |
| CC for partial structure (%) | 39.79 | - | - | - |
| **Refinement** |  |  |  |  |
| Resolution  (Å) ^1^ |  | 43.99-1.75 | 49.16-2.20 | 44.59-2.35 |
| Reflections (working/test)^1^ |  | 129,127/6,837 | 120,466/6,428 | 241,838/12,800 |
| *R*_factor_ / *R*_free_ (%)^1,3^ |  | 15.2/17.4 | 15.1/19.5 | 16.1/21.3 |
| No. of atoms  (protein/  NADPH/Zn^2+^/ Co^2+^/water) |  | 9,773/-/4/-/564 | 9,622/-/4/4/309 | 38,908/768/16/-/1,107 |
| **Model Quality** |  |  |  |  |
| R.m.s deviations |  |  |  |  |
| Bond lengths (Å) |  | 0.009 | 0.011 | 0.011 |
| Bond angles (^o^) |  | 1.009 | 1.032 | 1.172 |
| Average *B*-factor (Å^2^) |  |  |  |  |
| All Atoms |  | 27.8 | 37.9 | 43.4 |
| Protein |  | 27.4 | 37.9 | 43.5 |
| Zn^2+^ or Zn^2+^/ Co^2+^ |  | 20.1 | 41.3 | 37.2 |
| NADPH |  | - | - | 40.0 |
| Water |  | 33.3 | 37.2 | 29.7 |
| Coordinate error(maximum likelihood) (Å) |  | 0.17 | 0.21 | 0.27 |
| Ramachandran Plot |  |  |  |  |
| Most favored (%) |  | 98.1 | 98.4 | 97.4 |
| Additionally allowed (%) |  | 1.9 | 1.6 | 2.4 |

1. Values in parenthesis are for the highest resolution shell.
2. *R*_merge_ = ∑*_hkl_*∑*_i_* |*I_i_*(*hkl*) - <*I*(*hkl*)>| / ∑*_hkl_*∑*_i_* *I_i_*(*hkl*), where *I_i_*(*hkl*) is the intensity

measured for the *i*th reflection and <*I*(*hkl*)> is the average intensity of all reflections with indices hkl.

1. *R*_factor_ = ∑*_hkl_* ||*F*_obs_ (*hkl*) | - |*F*_calc_ (*hkl*) || / ∑*_hkl_* |*F*_obs_ (*hkl*)|; Rfree is calculated in an

identical manner using 5% of randomly selected reflections that were not included in the refinement.

1. *R*_meas_ = redundancy-independent (multiplicity-weighted) *R*_merge_[[1](#_ENREF_1),[2](#_ENREF_2)]. *R*_pim_ = precision-indicating (multiplicity-weighted) *R*_merge_[[3](#_ENREF_3),[4](#_ENREF_4)].
2. CC_1/2_ is the correlation coefficient of the mean intensities between two random half-sets of data [[5](#_ENREF_5),[6](#_ENREF_6)].

**Supplemental References**:

1. Evans PR (2011) An introduction to data reduction: space-group determination, scaling and intensity statistics. Acta Crystallogr D Biol Crystallogr 67: 282-292.

2. Evans P (2006) Scaling and assessment of data quality. Acta Crystallogr D Biol Crystallogr 62: 72-82.

3. Diederichs K, Karplus PA (1997) Improved R-factors for diffraction data analysis in macromolecular crystallography. Nat Struct Biol 4: 269-275.

4. Weiss MS (2001) Global indicators of X-ray data quality. Journal of Applied Crystallography 34: 130-135.

5. Karplus PA, Diederichs K (2012) Linking crystallographic model and data quality. Science 336: 1030-1033.

6. Evans P (2012) Biochemistry. Resolving some old problems in protein crystallography. Science 336: 986-987.
